# Neutrophil extracellular traps in patients with liver cirrhosis and hepatocellular carcinoma

**DOI:** 10.1038/s41598-021-97233-3

**Published:** 2021-09-09

**Authors:** Robin Zenlander, Sebastian Havervall, Maria Magnusson, Jennie Engstrand, Anna Ågren, Charlotte Thålin, Per Stål

**Affiliations:** 1 Department of Clinical Chemistry, Karolinska University Hospital, Stockholm, Sweden; 2 Department of Laboratory Medicine, Karolinska Institutet, Huddinge, Stockholm, Sweden; 3 Department of Medicine, Karolinska Institutet, Huddinge, Stockholm, Sweden; 4 Division of Gastroenterology, Department of Specialized Medicine, Danderyd Hospital, Stockholm, Sweden; 5 Department of Clinical Sciences, Karolinska Institutet Danderyd Hospital, Stockholm, Sweden; 6 Division of Pediatrics, CLINTEC, Karolinska Institutet, Stockholm, Sweden; 7 Astrid Lindgren Children’s Hospital, Karolinska University Hospital, Stockholm, Sweden; 8 Department of Molecular Medicine and Surgery, Karolinska Institutet, Stockholm, Sweden; 9 Coagulation Unit, Department of Hematology, Karolinska University Hospital, Stockholm, Sweden; 10 Division of Surgery, Department of Clinical Science, Intervention and Technology, Karolinska Institutet, Karolinska University Hospital, Stockholm, Sweden; 11 Department of Internal Medicine and Infectious Diseases, Danderyd Hospital, Stockholm, Sweden; 12 Division of Hepatology, Department of Upper GI Diseases, Karolinska University Hospital, Stockholm, Sweden

**Keywords:** Hepatocellular carcinoma, Biomarkers, Liver cancer, Liver cirrhosis

## Abstract

Neutrophil extracellular traps (NETs) are web-like structures consisting of DNA, histones and granule proteins, released from neutrophils in thrombus formation, inflammation, and cancer. We asked if plasma levels of the NET markers myeloperoxidase (MPO)-DNA and citrullinated histone H3 (H3Cit)-DNA, are elevated in liver cirrhosis and hepatocellular carcinoma (HCC) and if the levels correlate with clinical parameters. MPO-DNA, H3Cit-DNA, and thrombin–antithrombin (TAT) complex, as a marker of coagulation activity, were measured using ELISA in plasma from 82 patients with HCC, 95 patients with cirrhosis and 50 healthy controls. Correlations were made to clinical parameters and laboratory data and patients were followed for a median of 22.5 months regarding thrombosis development. H3Cit-DNA was significantly (p < 0.01) elevated in plasma from cirrhosis (66.4 ng/mL) and HCC (63.8 ng/mL) patients compared to healthy controls (31.8 ng/mL). TAT levels showed similar pattern (3.1, 3.7, and 0.0 µg/mL respectively, p < 0.01). MPO-DNA was significantly (p < 0.01) elevated in cirrhosis patients (0.53 O.D.) as compared to controls (0.33 O.D.). Levels of MPO-DNA and H3Cit-DNA correlated positively with Child–Pugh and MELD score. TAT was increased in all Child–Pugh and MELD groups. In multivariable logistic regression, Child B and C liver cirrhosis were independent predictors of elevated H3Cit-DNA in plasma. Levels of MPO-DNA and H3Cit-DNA were similar in patients with or without history of thrombosis, or thrombus formation during follow-up. In conclusion, plasma markers of NET formation are elevated in liver cirrhosis and correlate to the degree of liver dysfunction in patients with liver cirrhosis and/or HCC. The presence of HCC did not further increase the plasma levels of NET markers as compared to patients with cirrhosis only.

## Introduction

Neutrophils are the most abundant leucocyte in the human body and part of the innate immune system. They kill pathogens through phagocytosis and degranulation through the release of proteolytic and antimicrobial enzymes^1^. Neutrophils may also release DNA together with histones and other granule proteins, such as neutrophil elastase (NE) and myeloperoxidase (MPO), resulting in web-like structures, referred to as neutrophil extracellular traps (NETs)^2^. NETs have been shown to trap and neutralize invading pathogens^3^.

Chromatin decondensation is a crucial step in NET formation. The process is initiated through citrullination of positively charged histones by the calcium dependent enzyme peptidyl-arginine deiminase 4 (PAD4). Citrullination reduces the electrostatic force between histones and DNA, which leads to chromatin decondensation. Subsequent rupture of nuclear and cellular membranes results in the extracellular release of NETs^3^.

NETs can be demonstrated directly by conventional or electron microscopy^3^, or indirectly as circulating cell free DNA (cfDNA) or MPO-DNA^4^. However, cell-free DNA can be released from any type of cell damage and MPO-DNA can be detected after neutrophil activation without NET formation^4^. The detection of citrullinated histones, such as H3Cit, is considered a more specific marker for NET formation due to the critical role of histone citrullination in this process^3^.

Increased NET formation has been associated to several diseases and pathological conditions. In thrombus formation, NETs capture thrombocytes and erythrocytes, and provides a scaffold for the coagulation cascade^5^. NETs can propagate inflammation^6^, and increased formation has been demonstrated in autoimmune diseases, cancer, and atherosclerosis^3^. NETs produced during inflammation may promote cancer development^7^ and in tumor-bearing mice, NETs cause systemic inflammation and thrombosis^8^.

In a recent study on patients with hepatocellular carcinoma (HCC), isolated neutrophils displayed increased NET formation in vitro^9^. Elevated MPO-DNA was associated to increased mortality after liver surgery of primary liver cancer^10^. Patients with non-alcoholic steatohepatitis had elevated serum MPO-DNA, and in a mice model of HCC, inhibition of NET formation decreased tumor growth^6^.

The hemostatic situation in liver cirrhosis is complex with both an increased risk of bleeding and thrombosis^11,12^. The prothrombotic state could, at least partially, be explained by diminished production of anticoagulant factors such as protein C and S. However, decreased synthesis of other coagulation factors, overactivation of tissue plasminogen activator and the sequestration of platelets in the spleen would have an opposite effect^13^. Portal vein thrombosis is common in HCC^14,15^, which could be attributed to either the malignancy itself or to reduced portal blood flow due to increased vascular resistance^13,16^. A marker of coagulation activation is thrombin–antithrombin complex (TAT)^17,18^, which is increased in cirrhotic patients with portal vein thrombosis^19^.

H3Cit-DNA as a marker of NET formation has previously not been determined in patients with liver cirrhosis. In this study, we determined NET formation in patients with liver cirrhosis with or without HCC, using both MPO-DNA and a recently developed and well-validated H3Cit-DNA assay^4^. We asked whether plasma levels of NET markers would correlate to the presence of HCC, tumor burden, liver function, thrombus formation, inflammatory parameters, or underlying liver disease.

## Methods

### Patients

Patients with liver cirrhosis or HCC (with or without cirrhosis) at the Department of Upper Gastrointestinal Diseases at Karolinska University Hospital, a tertiary hospital in Stockholm, Sweden, were eligible to be included in the study. Exclusion criteria were age < 18 years, tumors not fulfilling radiological criteria according to guidelines from the European Association for the Study of the Liver (EASL)^20^ or other concurrent malignancies than HCC. Patients were included from April 11, 2013 to October 29, 2019. Patients were followed-up regarding development of thrombi in liver vessels until death, loss of follow-up or end of study. Cirrhosis patients underwent follow-up regarding HCC development. All included patients provided written informed consent. The study was approved by The Swedish Ethical Review Authority (EPM 2019-05680) and was conducted in accordance with the Declaration of Helsinki.

The diagnosis of liver cirrhosis was based on at least one of the following: (a) histologic criteria from liver biopsy, (b) transient elastography > 15 kPa together with laboratory values consistent with chronic liver disease^21^ or (c) imaging findings demonstrating portal hypertension and irregular liver parenchyma. All patients in the cirrhotic non-HCC group had undergone imaging with ultrasound or computerized tomography of the liver within 3 months prior to inclusion.

In patients with cirrhosis, the Child–Pugh score and Model of End-Stage Liver Disease (MELD) score were calculated at inclusion. In cases where HCC occurred in a liver without cirrhosis, Child–Pugh score was considered Child–Pugh A.

In cirrhotic patients with HCC, the diagnosis of HCC was based on radiological criteria according to guidelines from EASL^20^. In non-cirrhotic livers, HCC diagnosis was based on histologic criteria from tumor biopsy.

Medical charts were examined regarding clinical data which included age, sex, BMI, underlying liver diseases (non-alcoholic steatohepatitis, autoimmune hepatitis, viral hepatitis, alcoholic liver disease, primary biliary cholangitis, cryptogenic cirrhosis, hemochromatosis, Wilson disease, congenital liver fibrosis, Budd–Chiari syndrome, methotrexate-induced liver cirrhosis), diabetes mellitus, laboratory results (hemoglobin, platelets, leucocytes, bilirubin, PT (INR), C-reactive protein (CRP), albumin), Child–Pugh score, presence of ascites or esophageal varices, history of thrombosis in liver vessels, macrovascular invasion, lymph node invasion, distant metastases, tumor size, number of tumors, active treatment against HCC and survival. Alcohol overconsumption was defined as reporting an intake ≥ 30 g per day (or ≥ 14 units per week) for males or ≥ 20 g per day (≥ 10 units per week) for females, or having a diagnosis of alcoholic liver disease in medical charts. Performance status according to The Eastern Cooperative Oncology Group (ECOG) was registered in HCC patients at the time of blood sampling.

### Healthy controls

Plasma samples from 50 sex and age matched healthy individuals were obtained from controls included in a previous study investigating NETs in cancer patients^22^. The group comprised of 66% men (n = 33) and the median age was 68 (IQR 56–71). Healthy controls had no active or previous cancer diagnosis and no history of previous liver disease or other comorbidities other than hypertension. No regular medication other than antihypertensive treatment was allowed.

### Sampling and analysis of NET markers

At inclusion, blood was drawn into EDTA (ethylenediaminetetraacetic acid) tubes using standard procedure. The tubes were centrifuged at 2000 G for 10 min within one hour from sampling before plasma was separated. Plasma was then frozen at − 80 °C until analysis.

H3Cit-DNA complexes were measured according to previous published ELISA-based protocol^23^. In brief, microtiter plates were coated with 50 µL of 5 µg/mL anti-histone H3 (citrulline R8) antibody (Cat# 232939, Abcam, Cambridge, United Kingdom) overnight and then blocked with a 1% BSA solution in PBS. The plasma samples were then added together with a monoclonal anti-DNA antibody (Cell Death ELISAPLUS, Roche, Basel, Switzerland) as the detection antibody. An automatic plate reader measured the optical density at a wavelength of 650 nm. The standard curve was generated from semi-synthetic recombinant nucleosomes (Cat#16-1362, EpiCypher, Durham, North Carolina, United States).

MPO-DNA complexes were quantified using a previously described capture ELISA^24^ using a monoclonal MPO antibody (Cat# 0400-0002, ABD Serotec, Oxford, United Kingdom) for capture and a monoclonal anti-DNA antibody (Cell Death Detection ELISA PLUS kit, Cat# 11 774425001, Roche, Basel, Switzerland) for detection.

TAT complexes were analyzed using the Enzygnost TAT micro ELISA (Siemens, Munich, Germany), also according to the manufacturers protocol.

### Statistics

Baseline data for the patients was analyzed using descriptive statistics and presented as median together with interquartile range (IQR) for continuous variables and frequency together with percentage for categorical variables. Comparison between groups for continuous variables were analyzed using Mann–Whitney U test and for categorical variables Pearson's Chi squared test. The correlation between levels of NET markers and lab parameters were estimated using linear regression and presented as R^2^. Survival was calculated from the time of blood sampling to death or censoring at end of follow-up October 20, 2020 (whichever occurred first), and evaluated with Kaplan–Meier curves. Univariable and multivariable logistic regression models were used to analyze factors associated with a high level of H3Cit-DNA (dichotomized into above or below 200 ng/mL). A p value below 0.05 was considered to be significant. All analyses were performed in Graphpad Prism (version 5.04), RStudio (version 1.1.453) or STATA version 15.0 (StataCorp, Collage Station, Texas, USA).

## Results

### Baseline characteristics of patients

A total of 227 participants were enrolled, consisting of 82 patients with HCC (of which 67 also had liver cirrhosis), 95 patients with cirrhosis without HCC, and 50 healthy controls. The baseline data of the patients with HCC and those with cirrhosis are summarized in Table 1. The HCC patients were older than the cirrhosis patients (median 69.0 years vs 63.4 years) and with a higher proportion of males (83% vs 62%). No significant difference was found in Child–Pugh or MELD score between the two patient groups. Ascites, history of thrombosis and inflammatory markers such as CRP and leukocytes were similar between the groups, whereas esophageal varices was slightly more frequent in the cirrhosis group.

**Table 1:** Baseline data of patients with HCC or cirrhosis (without HCC).

	HCC (n = 82)		Cirrhosis (n = 95)		p value
	Median/number (%)	IQR	Median/number (%)	IQR	
Age (years)	69.0	(63.1–74.8)	63.4	(56.3–70.4)	** < 0.01**
Sex (male)	68 (83%)		62 (65%)		**0.01**
BMI	27.1	(24.8–30.0)	27.4	(24.0–31.5)	0.83
Diabetes	36 (44%)		34 (36%)		0.34
Child﻿ Pugh					
- A	45 (55%)		47 (49%)		0.29
- B	31 (38%)		34 (36%)		
- C	6 (7%)		14 (15%)		
MELD	9	(7–12)	10	(8–13)	0.19
Etiolo﻿gy					
- NASH	15 (18%)		19 (20%)		**0.05**
- Viral hepatitis	31 (38%)		20 (21%)		
- Alcohol	17 (21%)		34 (36%)		
- Other	19 (23%)		22 (23%)		
Esophageal varices	38 (46%)		62 (65%)		**0.02**
Ascites	29 (35%)		44 (46%)		0.19
CRP (mg/L)	6	(2–16)	4	(1–8)	**0.02**
Leucocytes (10^9^/L)	6.1	(5.1–7.6)	5.4	(4.2–6.7)	**< 0.01**
Platelets (10^9^/L)	186	(125.5–248.5)	126.5	(92.3–179.8)	**< 0.01**
Hemoglobin (g/L)	133.5	(121.5–143.2)	131	(117–145)	0.75
Bilirubin (µmol/L)	13	(9–21)	17	(11–34)	**0.01**
PT (INR)	1.2	(1.1–1.4)	1.2	(1.1–1.4)	0.08
Albumin (g/L)	33	(29–36)	33	(29–37)	0.95
Thrombi (liver)^a^	10 (12%)		13 (14%)		0.94
Thrombi (all)^b^	11 (13%)		17 (18%)		0.94
Tumor ≥ 8 cm	15 (18%)		–		
Total no. of tumors > 3	29 (35%)		–		
MVI or EHS^c^	31 (38%)		–		

^a^History of thrombi in liver vessels.

^b^History of thrombi, any vessel.

^c^Macrovascular invasion or extrahepatic spread at time of inclusion. Extrahepatic spread includes lymph node invasion and distant metastases.

### Plasma H3Cit-DNA, MPO-DNA and TAT complex levels in relation to the presence of HCC

**Figure 1:**
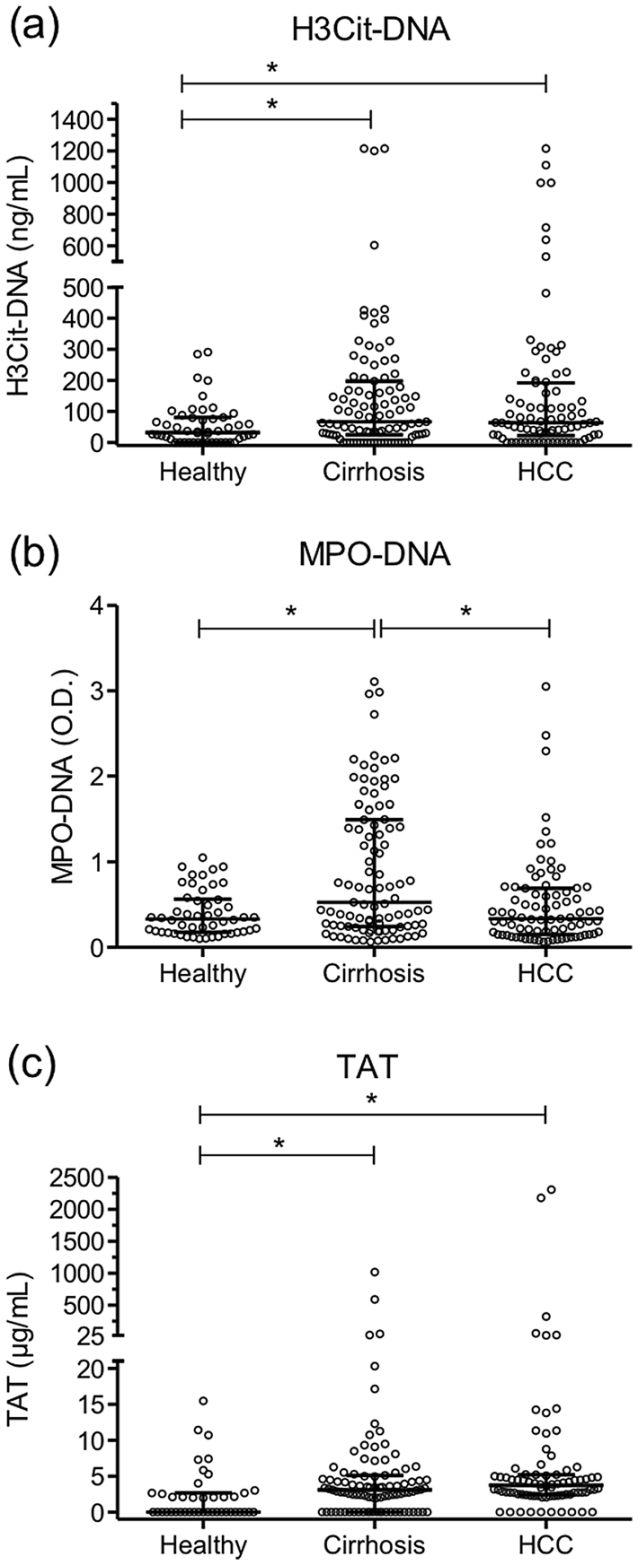
Plasma levels of H3Cit-DNA, MPO-DNA and TAT in patients with cirrhosis, HCC and in healthy controls. (**a**) Plasma H3Cit-DNA was significantly elevated in both the HCC and cirrhosis group as compared to healthy controls. (**b**) Plasma MPO-DNA was significantly increased in the cirrhosis group as compared to healthy controls and the HCC group. (**c**) Plasma TAT was significantly elevated in both the HCC and cirrhosis group as compared to healthy controls. Figures were created using GraphPad Prism version 5.04 for Windows, GraphPad Software, San Diego, California, USA, www.graphpad.com.

Plasma H3Cit-DNA levels were significantly elevated in both HCC and cirrhosis patients compared to healthy controls, and without difference between the latter two patient groups (Fig. 1a). MPO-DNA was significantly increased in cirrhotic patients in comparison to healthy controls or HCC patients (Fig. 1b). TAT-complex levels were similar in patients with HCC or cirrhosis, but significantly higher than in healthy controls (Fig. 1c).

### Plasma H3Cit-DNA, MPO-DNA and TAT complex levels in relation to liver function

**Figure 2:**
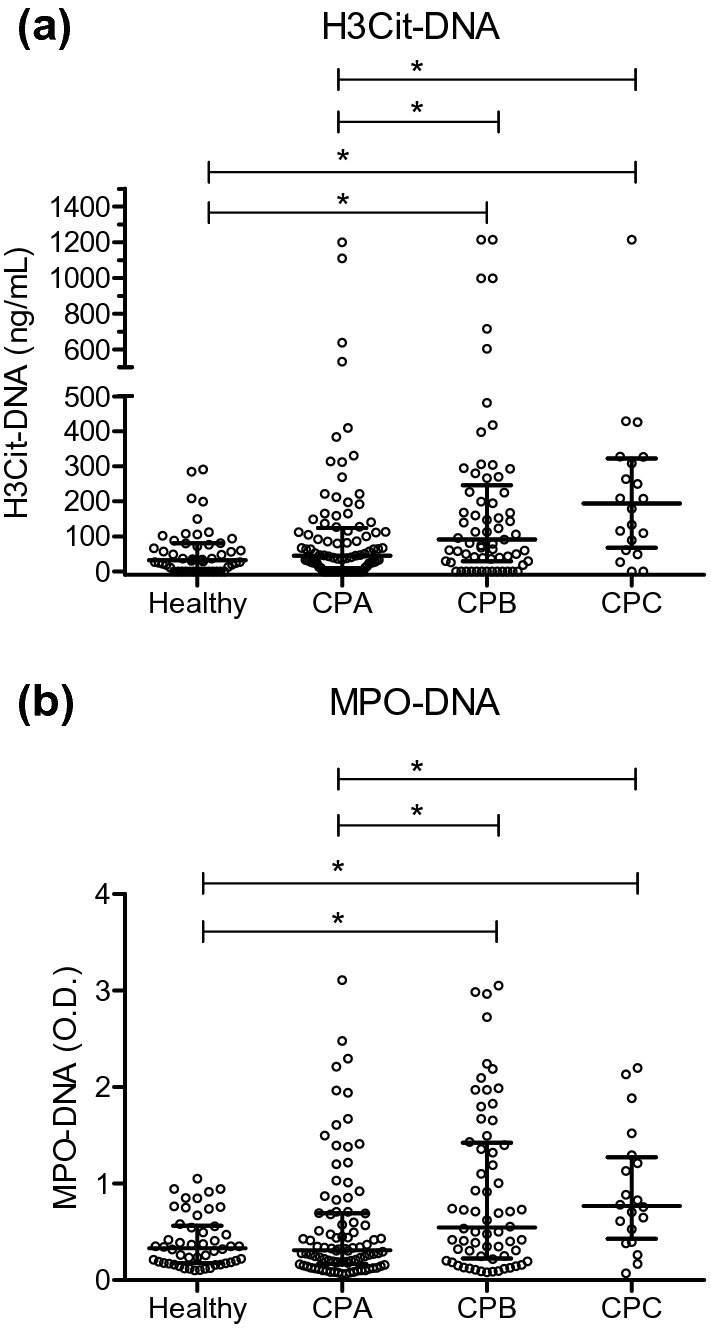
Plasma levels of H3Cit-DNA and MPO-DNA in healthy controls and patients with different Child–Pugh Scores. The cirrhosis and HCC groups are pooled together. (**a**) Child–Pugh B (CPB) and Child–Pugh C (CPC) patients had significantly elevated H3Cit-DNA levels as compared to healthy controls and Child–Pugh A (CPA). The difference between Child–Pugh B and Child–Pugh C was not significant. (**b**) Child–Pugh B and Child–Pugh C had significantly elevated MPO-DNA levels as compared to healthy controls and Child–Pugh A. The difference between Child–Pugh B and Child–Pugh C was not significant. Figures were created using GraphPad Prism version 5.04 for Windows, GraphPad Software, San Diego, California, USA, www.graphpad.com.

Figure 2 demonstrates plasma H3Cit-DNA and MPO-DNA in patients with different Child–Pugh scores. For H3Cit-DNA (Fig. 2a) and MPO-DNA (Fig. 2b), an increase was seen from Child–Pugh A to Child–Pugh C, where Child–Pugh A had levels similar to those of healthy controls. A similar pattern with higher H3Cit-DNA and MPO-DNA levels in Child–Pugh B or C patients compared to Child–Pugh A patients was also seen when only comparing patients with cirrhosis (supplementary Figure S1). No difference was seen when comparing HCC patients without cirrhosis to HCC patients with Child–Pugh A cirrhosis (supplementary Table S1). H3Cit-DNA levels were significantly higher in patients with a MELD score ≥ 14 as compared with those < 14 (Table 2). The same was also seen for MPO-DNA (Table 2). Plasma TAT levels did not correlate to liver function measured as Child–Pugh score.

**Table 2:** H3Cit-DNA levels (ng/mL) and MPO-DNA levels (O.D.) in patients stratified on various clinical variables.

	H3Cit-DNA (ng/mL)		p value	MPO-DNA (O.D.)		p value
	Group 1 median (IQR)	Group 2 median (IQR)		Group 1 median (IQR)	Group 2 median (IQR)	
**All patients**						
MELD (< 14 vs ≥ 14)	57.2 (13.2–142.2)	173.5 (51.7–296.5)	** < 0.01**	0.35 (0.19–0.87)	0.71 (0.39–1.47)	**0.02**
Etiology (HepC vs NASH)	39 (12.6–119.5)	110.5 (34.1–243.6)	**0.01**	0.23 (0.12–0.49)	0.76 (0.39–1.38)	** < 0.01**
Ascites (no vs yes)	57.2 (24.3–159.1)	90.9 (25.3–224.9)	0.21	0.33 (0.18–0.72)	0.64 (0.26–1.2)	** < 0.01**
Alcohol overconsumption^a^ (no vs yes)	67 (24.8–196.1)	61.7 (5.7–150.1)	0.74	0.38 (0.18–0.86)	0.57 (0.3–1.28)	**0.03**
Esophageal varices (no vs yes)	62.7 (15.1–139.7)	83.1 (26–214.4)	0.29	0.32 (0.16–0.69)	0.56 (0.25–1.24)	** < 0.01**
Diabetes (no vs yes)	63 (15.4–162)	84.6 (28.1–205.4)	0.44	0.41 (0.19–0.9)	0.43 (0.22–1.08)	0.68
BMI (< 30 vs ≥ 30)	63.2 (10.2–195.8)	79.5 (29.7–172.1)	0.38	0.42 (0.19–0.9)	0.44 (0.26–1.39)	0.36
History of thrombosis in liver vessels (no vs yes)	62.9 (25–193.9)	90.9 (0–143)	0.87	0.42 (0.2–0.92)	0.39 (0.17–1.45)	0.82
Develops thrombosis in liver vessels during follow-up (no vs yes)^b^	63.2 (25.0–198.4)	50.8 (25.9–92.4)	0.30	0.44 (0.20–0.91)	0.34 (0.20–1.15)	0.59
History of thrombosis in all vessels (no vs yes)	55.4 (18.3–147.6)	99.8 (0.0–236.1)	0.32	0.38 (0.19–0.77)	0.39 (0.17–1.45)	0.50
Develops thrombosis in any vessel during follow-up (no vs yes)^b^	56.2 (18.2–151.6)	42.5 (24.7–91.5)	0.49	0.38 (0.19–0.76)	0.38 (0.22–1.13)	0.89
**HCC patients only**						
Largest tumor (< 8 cm vs ≥ 8 cm)	50.3 (11.3–136.8)	112.6 (65.5–392.7)	**0.01**	0.33 (0.15–0.62)	0.53 (0.24–0.92)	0.09
Invasive HCC^c^ (no vs yes)	63.4 (28.5–193)	67 (0–119.7)	0.37	0.33 (0.16–0.7)	0.35 (0.15–0.63)	0.97
Tumor numbers (< 3 vs ≥ 3)	51.2 (17.1–111.8)	91.5 (39–292.5)	0.10	0.33 (0.15–0.64)	0.35 (0.17–0.59)	0.86
**Cirrhosis patients only**						
Develops HCC during follow-up (no vs yes)	81.3 (24.7–197.3)	30.9 (27.7–115.4)	0.52	0.53 (0.25–1.5)	0.71 (0.1–1.38)	0.42

Median follow-up for the evaluation of thrombosis development was 22.5 months, and 26 months for HCC development in patients with cirrhosis.

^a^Alcohol overconsumption defined as ≥ 14 units/week (males) or ≥ 10 units/week (females) or diagnosis of alcoholic liver disease in medical charts.

^b^Patients with a history of same type thrombosis are excluded.

^c^Macrovascular invasion or extrahepatic spread at time of inclusion. Extrahepatic spread includes lymph node invasion and distant metastases.

### Plasma H3Cit-DNA, MPO-DNA and TAT in relation to clinical parameters

Comparing H3Cit-DNA with MELD as a continuous variable, even though slope was significantly differed from 0, an R^2^ value of 0.02 implies only a weak correlation. No correlation was seen between H3Cit-DNA and platelet count (R^2^ = 0.02), PT-INR (R^2^ = 0.00), leucocyte count (R^2^ = 0.00), C-reactive protein (CRP) (R^2^ = 0.02) or bilirubin (R^2^ = 0.00). Similar results were seen with MPO-DNA. MPO-DNA was significantly higher in patients with ascites (p < 0.01) or esophageal varices (p < 0.01) as compared to patients without these clinical features. Patients with an underlying NASH had significantly higher H3Cit-DNA and MPO-DNA levels compared to those with viral hepatitis (Table 2). TAT levels were not correlated to any of these clinical parameters. Forty-eight patients had hepatitis C, 17 of whom had sustained virological response (SVR) after antiviral treatment. The H3Cit-DNA levels were similar in those with SVR (40.7 ng/mL) as in those with viremia (29.9 ng/mL). Only three patients had chronic hepatitis B, of which one was viremic. This patient had a high H3Cit-DNA of 1110 ng/mL whereas the two non-viremic HBV patients had levels of maximum 221 ng/mL.

One of the 177 patients in the cohort had dialysis at inclusion, with H3Cit-DNA level of 1214 ng/mL and MPO-DNA level of 2.98 O.D. Excluding this patient did not change the statistical significance in any of the analyses.

In HCC patients, H3Cit-DNA levels were significantly higher in patients with tumors ≥ 8 cm but similar in those with or without macrovascular invasion (MVI) or extrahepatic spread (EHS) (Table 2). Treatment with the multikinase inhibitor sorafenib (Nexavar^®^) was present at inclusion in 5 of 82 patients with HCC with no significant difference in H3Cit-DNA levels.

TAT levels were similar in patients with a history of liver vessel thrombosis (4.5 µg/mL vs 3.1 µg/mL, p = 0.07) compared to those without. However, TAT was significantly elevated in patients with a history of venous thrombosis in any vessel compared to patients without (4.2 µg/mL vs 2.7 µg/mL, p = 0.01). No differences were seen for H3Cit-DNA and MPO-DNA (see Table 2). The levels of TAT, H3Cit-DNA and MPO-DNA were not significantly different in patients who developed venous thrombosis in liver vessels or in any vessels as compared to those without thrombus development (Table 2, data not shown for TAT).

Patients were followed-up for a median of 22.5 months regarding the development of liver vessel thrombosis. Patients with or without liver vessel thrombosis development during follow-up had similar levels of TAT (3.2 µg/mL vs 3.4 µg/mL, p = 0.60), H3Cit-DNA (59.0 ng/mL vs 65.4 ng/mL, p = 0.43) and MPO-DNA (0.38 O.D. vs 0.43 O.D., p = 0.85).

Cirrhosis patients were followed regarding HCC development. During a median follow-up of 26 months, 6/95 (6.3%) of the cirrhosis patients developed HCC. Levels of H3Cit-DNA or MPO-DNA were not significantly different in those with or without HCC development (Table 2).

### Comparison of patients with H3Cit above or below 200 ng/mL (Table 3)

**Table 3:** Baseline data of the combined cohort of HCC and cirrhosis patients as divided into high or low levels of H3Cit-DNA. H3Cit-DNA of 200 ng/mL equals the 95^th^ percentile of H3Cit-DNA in healthy controls.

	H3Cit ≥ 200 (n = 41)		H3Cit < 200 (n = 136)		p value
	Median/number (%)	IQR	Median/number (%)	IQR	
Age (years)	66.2	(59.4–71.7)	66.4	(58.5–72.5)	0.58
Sex (male)	26 (63%)		104 (76%)		0.14
BMI	26.8	(24.4–30.6)	27.5	(24.2–31.3)	0.43
Diabetes	18 (44%)		52 (38%)		0.64
Child Pugh					
- A	13 (32%)		79 (58%)		** < 0.01**
- B	18 (44%)		47 (35%)		
- C	10 (24%)		10 (7%)		
MELD	11	(8–15)	9	(7–12)	** < 0.01**
Esophageal varices	28 (68%)		72 (53%)		0.12
Ascites	21 (51%)		52 (38%)		0.19
CRP (mg/L)	8	(3–19)	4	(1–9)	** < 0.01**
Leucocytes (10^9^/L)	6.2	(4.2–7.6)	5.7	(4.6–7.3)	0.41
Platelets (10^9^/L)	130	(84–224)	155	(103–208)	0.64
Bilirubin (µmol/L)	18	(12–38)	14	(9–23)	**0.02**
PT (INR)	1.3	(1.1–1.4)	1.2	(1.1–1.3)	**0.04**
Albumin (g/L)	31	(24–34)	33	(30–37)	** < 0.01**
Thrombi (liver)^a^	5 (12%)		18 (13%)		0.99
Thrombi (all)^b^	8 (20%)		20 (15%)		0.99
**HCC**	18 (44%)		64 (47%)		0.86
Tumor > 8 cm	6 (33%)		9 (14%)		0.15
MVI or EHS^c^	7 (39%)		24 (38%)		0.99

^a^History of thrombi in liver vessels.

^b^History of thrombi, any vessel.

^c^Macrovascular invasion or extrahepatic spread at time of inclusion. Extrahepatic spread includes lymph node invasion and distant metastases.

A reference interval for H3Cit was calculated for healthy subjects by using the 95th percentile giving a value of 203 ng/mL, which was approximated to 200 ng/mL. The patient cohort (n = 177) was stratified in H3Cit-DNA above or below 200 ng/mL. Liver function (Child–Pugh and MELD) was significantly more impaired and CRP higher in patients with H3Cit-DNA above 200 ng/mL. Kaplan–Meier analysis of overall survival (OS) was not significantly different between cirrhosis patients with H3Cit below and above 200 ng/mL, with 2-year OS of 91.2% and 79.1%, respectively (p = 0.62). Survival analysis was not performed for the HCC group due to the heterogeneity regarding treatment received.

Multivariable logistic regression (Table 4) showed Child–Pugh score to be significantly associated to H3Cit-DNA levels above or below 200 ng/mL when adjusted for etiology, CRP and HCC.

**Table 4:** Univariable and multivariable logistic regression on factors associated with H3Cit-DNA above or below 200 ng/mL.

	Univariable		Multivariable	
	Odds ratio (CI)	p value	Odds ratio (CI)	p value
Age > 65 years	0.98 (0.49–2)	0.95	–	–
Male sex	0.53 (0.25–1.14)	0.1	–	–
CRP > 5	1.95 (0.96–4.09)	0.07	1.48 (0.69–3.24)	0.32
Underlying liver disease				
- Hepatitis C	Ref		Ref	
- NASH	3.01 (1.04–9.18)	0.04	2.29 (0.74–7.41)	0.15
- Other	2.1 (0.86–5.65)	0.12	1.45 (0.56–4.18)	0.46
Child Pugh score				
- Child A	Ref		Ref	
- Child B or C	2.99 (1.45–6.43)	0.004	2.72 (1.27–6.07)	0.01
Diagnosis				
- Cirrhosis	Ref		Ref	
- HCC	0.88 (0.43–1.77)	0.72	0.96 (0.44–2.07)	0.92

## Discussion

This is the first evaluation of MPO-DNA and H3Cit-DNA in plasma from patients with liver cirrhosis and various degrees of liver dysfunction (Child–Pugh A to C), with or without concurrent HCC. Increased formation of NETs has been found in various chronic inflammatory diseases and in malignancies and was shown to correlate to thrombus formation, inflammation, and malignant progression^3,5,8,25–27^. It is unknown whether an increased NET formation contributes to thrombus formation and malignant progression of liver cirrhosis and HCC. The first step to elucidate a putative association is to investigate NET formation in patients with various degree of chronic end-stage liver disease. We therefore determined markers of NETs in plasma of patients with liver cirrhosis and HCC both with MPO-DNA, a standard method in use for several years, and the recently developed H3Cit-DNA assay.

Our main findings are that both MPO-DNA and H3Cit-DNA are elevated in plasma from patients with end-stage liver cirrhosis (Child–Pugh B and C, or MELD > 14), whereas Child–Pugh A patients (and HCC patients without cirrhosis) with preserved liver function have levels similar to those of healthy controls. These findings indicate that increased NET formation is a late feature of cirrhosis progression, possibly associated to the more pronounced systemic inflammatory state seen in decompensated cirrhosis^28^. Patients with high H3Cit-DNA levels (> 200 ng/mL) had slightly higher C-reactive protein (CRP) levels in plasma than those with levels < 200 ng/mL, but we found no strict correlation between CRP and H3Cit-DNA in linear regression analysis. However, CRP is a poor indicator of inflammation in advanced liver disease since its production may be hampered by the liver dysfunction.

Our results agree with previous publications such as Blasi et al.^29^ who have showed that markers of NET are elevated in patients with cirrhosis as compared to healthy controls. However, in contrast to our findings, Agraz-Cibrián et al. showed that neutrophils from ascites fluid from cirrhotic patients with spontaneous bacterial peritonitis had reduced ability to form NETs^30^. The same author also showed that neutrophils from peripheral blood of cirrhosis patients also had reduced ability of NET formation upon stimulation in vitro^31^. The results from Agraz-Cibrián in combination with those of the present study indicate that although neutrophils in decompensated liver cirrhosis have a reduced capacity of NET formation upon maximal ex-vivo stimulation, there is still an elevation of NET markers in the circulation of decompensated cirrhosis patients, possibly due to a sustained chronic systemic inflammation in advanced chronic liver disease.

Unexpectedly, we found that the presence of HCC did not further increase the plasma levels of MPO-DNA or H3Cit-DNA as compared to the corresponding patients with cirrhosis only. As seen in Table 2, patients with large tumors (≥ 8 cm in diameter) expressed elevated H3Cit-DNA levels, however this could not be confirmed as an independent predictor in multivariable analysis. In contrast to our results, Yang and co-workers found NET formation to be elevated in HCC patients in general. In their in vitro studies, MPO-DNA could trap HCC cells, induce cell-death resistance, and promote metastatic disease^9^. However, Yang et al. isolated granulocytes from HCC patients and detected NETs production in vitro, whereas we analyzed the presence of markers of NETs in the peripheral circulation. Interestingly, Yang et al. detected NET markers in tumor specimens, indicating a local NET formation in proximity to a malignant lesion. If such lesions are small, levels of NET markers would possibly be below the detection threshold in peripheral blood, whereas large tumors, possibly indicated in our study, would give rise to elevated markers of NET formation in plasma. Taken together, our results imply that in patients with HCC, NET formation is not as extensive as in advanced cirrhosis with liver dysfunction, however a local NET formation adjacent to a HCC tumor cannot be fully ruled out.

In the univariable logistic regression we found that an underlying non-alcoholic steatohepatitis (NASH) was associated with higher plasma H3Cit-DNA levels while a viral hepatitis etiology was not. This association did not remain in the multivariable regression model, possibly suggesting no independent association between NASH and NETs. However, previous studies by van der Windt and co-workers found elevated markers of NETs in patients with NASH. In a mice model of steatosis and HCC, NET formation paralleled inflammation and increased tumor formation^6^. In the model, inhibition of NET formation by DNase or by using PAD4 knock-out (PAD4^−/−^) mice reduced both inflammation and tumor development. NASH is associated to the metabolic syndrome, displaying a chronic low-grade inflammation^32^ and an increased risk for HCC development^33^. Although not confirmed in our study, these findings point at a possible association which warrants further evaluation.

The association of NETs with thrombus formation has long been established. NETs can promote venous thrombosis by acting as a scaffold for platelets, red blood cells and procoagulant molecules within the vessel, or by activating factor XII in the coagulation cascade^27^. NET formation may play an important role in cancer-associated thrombosis. In animal models, tumor cells and cytokines such as granulocyte colony-stimulating factor and IL-8 induce NETs^25^. Portal vein thrombosis (PVT) is a complication commonly associated with liver cirrhosis and HCC that may worsen the patient’s prognosis^34^. The prevalence of PVT is up to 23% in cirrhotic patients waiting for liver transplantation^35^, 30% among HCC patients with cirrhosis, and 11% in non-cirrhotic HCC patients^15^. In a small unblinded study, enoxaparin (a low molecular weight heparin) had a protective effect on cirrhosis decompensation and improved survival^36^. Heparins are negatively charged molecules that have been suggested to disrupt NETs^5^, and low molecular weight heparins have also been shown to inhibit NET formation^37^. Thus, increased NET formation could be associated to hypercoagulability manifested as portal thrombosis and development of sinusoidal micro-thrombi in end-stage liver disease. As expected, we found that TAT, a marker of intravascular hypercoagulability, was elevated in both patients with cirrhosis and patients with HCC as compared with healthy controls^19^. However, there was a large variation in the patient groups, with many patients having normal levels comparable to those of healthy controls. We found no correlation between TAT and H3Cit-DNA or MPO-DNA, indicating different pro-coagulation mechanisms by TAT production and NET formation. Furthermore, we found no correlation between H3Cit-DNA levels, MPO-DNA levels or TAT levels and previous history of portal thrombosis, or to later development of thrombosis within the follow-up period. Thus, in this setting, neither H3Cit-DNA nor MPO-DNA could act as a biomarker for previous or future risk of thrombus formation.

A possible explanation for the lack of association between NET formation and liver vessel thrombosis could be that other factors play important roles for thrombus formation in liver cirrhosis. In a prospective study by Zanetto et al.^38^, the authors showed that cirrhotic patients with HCC who developed PVT, when studying their whole blood clot initiation using rotational thromboelastometry (ROTEM), had a higher level of fibrinogen function which could explain the increased risk of PVT in this patient group. Also, cirrhotic patients with HCC who developed portal vein thrombosis were shown to have an increased level of annexin V and endothelial-derived microparticles^39^, indicating a role for microparticles in thrombus formation in severe liver disease.

The strengths of the current project are the use of the novel, validated and specific H3Cit-DNA assay to detect NETs, the mapping of a well-characterized patient cohort with both cirrhosis and HCC, and the associations to different degrees of liver dysfunction. To our knowledge, the correlation of NET markers to different stages of liver cirrhosis (Child–Pugh A to C) has not previously been studied. The use of H3Cit-DNA instead of the previously more commonly used MPO-DNA provides a new insight into NET formation in cirrhosis by increasing specificity, since MPO-DNA could in theory form independently of NET formation. The major limitation is that this study is observational and thus cannot reveal the underlying mechanisms for increased thrombosis formation and liver cirrhosis progression in end-stage liver disease and the role of NETs in that setting. We consider these findings as a base for further prospective studies on the role of NET formation in the development of portal thrombosis and progression of liver dysfunction in patients with liver cirrhosis. As previously mentioned, heparins and low molecular weight heparins have been shown to interfere with NET formation and enoxaparin has been shown to have a potential protective effect in cirrhotic patients. Future research should, in addition to heparins, also focus on the applicability of other therapeutics that inhibit or dissolve NET formation, such as DNAses and peptidyl arginine deiminase type IV (PAD-4) inhibitors.

In conclusion, we have demonstrated that H3Cit-DNA, a specific marker of NET formation, is elevated in plasma of patients with liver cirrhosis and impaired liver function, with a correlation to the degree of liver dysfunction. The presence of HCC did not further increase the plasma levels of NET markers as compared to corresponding patients with cirrhosis only. Although associated to portal thrombosis in previous studies, NETs levels in plasma could not act as a biomarker for previous or future risk of thrombosis in this setting.

## Supplementary Information

Supplementary Information 1.

## Data Availability

The datasets generated and/or analyzed during the current study are available from the corresponding author on reasonable request.

## Abbreviations

BCLC: Barcelona Clinic Liver Cancer
BMI: Body mass index
cfDNA: Cell free DNA
CRP: C-reactive protein
EHS: Extrahepatic spread
ECOG: The Eastern Cooperative Oncology Group
EASL: European Association for the Study of the Liver
EDTA: Ethylenediaminetetraacetic acid
H3Cit: Citrullinated histone H3
HCC: Hepatocellular carcinoma
IQR: Interquartile range
MELD: Model for End-Stage Liver Disease
MVI: Macrovascular invasion
MPO: Myeloperoxidase
NE: Neutrophil elastase
NETs: Neutrophil extracellular traps
O.D.: Optical density
OS: Overall survival
PAD4: Peptidyl-arginine deiminase 4
PT (INR): Prothrombin time international normalized ratio
PVT: Portal vein thrombosis
TAT: Thrombin–antithrombin
