# Supplementary material for: Neutrophil extracellular traps in patients with liver cirrhosis and hepatocellular carcinoma
Source: Sci Rep. 2021 Sep 9;11:18025. doi: 10.1038/s41598-021-97233-3 (PMC8429678; doi:10.1038/s41598-021-97233-3)
Supplement: Supplementary file 1 — Supplementary Information 1. [file 41598_2021_97233_MOESM1_ESM.pdf]

Supplementary Information for

Neutrophil extracellular traps in patients with liver cirrhosis and hepatocellular carcinoma

Robin Zenlander* [1,2,3], Sebastian Havervall[4,5], Maria Magnusson[6,7,8,9], Jennie Engstrand[10], Anna Ågren[5,8,9], Charlotte Thålin[5,,11], Per Stål[3,12]

[1] Department of Clinical chemistry, Karolinska University Hospital, Stockholm, Sweden

[2] Department of Laboratory Medicine, Huddinge, Karolinska Institutet, Stockholm, Sweden

[3] Department of Medicine, Huddinge, Karolinska Institutet, Stockholm, Sweden

[4] Department of specialized medicine, Division of gastroenterology, Danderyd Hospital, Stockholm, Sweden.

[5] Department of Clinical Sciences, Karolinska Institutet Danderyd Hospital, Stockholm, Sweden

[6] Division of Pediatrics, CLINTEC, Karolinska Institutet, Stockholm, Sweden

[7] Astrid Lindgren Children's Hospital, Karolinska University Hospital, Stockholm, Sweden

[8] Department of Molecular Medicine and Surgery, Karolinska Institutet, Stockholm, Sweden

[9] Coagulation Unit, Department of Hematology, Karolinska University Hospital, Stockholm, Sweden

[10] Division of Surgery, Department of Clinical Science, Intervention and Technology, Karolinska Institutet, Karolinska University Hospital, Stockholm, Sweden

[11] Department of Internal medicine and Infectious diseases, Danderyd Hospital, Stockholm, Sweden

[12] Division of Hepatology, Department of Upper GI diseases, Karolinska University Hospital, Stockholm, Sweden

Correspondence and reprint requests:

Robin Zenlander, Department of Laboratory Medicine, Huddinge, Karolinska Institutet, Stockholm, Sweden

Phone: +46 (0) 8 585 812 45, Fax: +46 (0) 8 585 812 60

E-mail: robin.zenlander@ki.se

Supplementary Figure S1

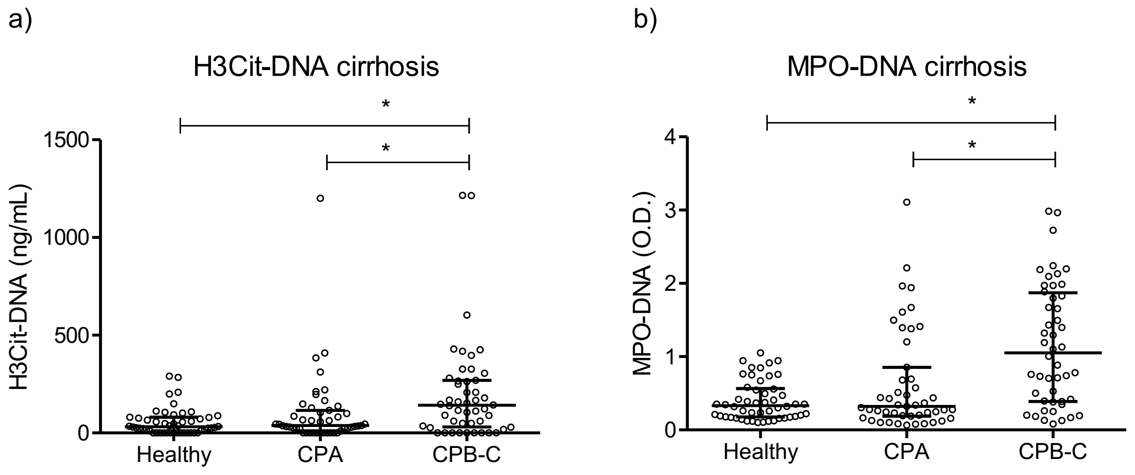

Supplementary Figure S1: Plasma levels of H3Cit-DNA and MPO-DNA in healthy controls and patients with different Child-Pugh scores. **a)** Patients with Child-Pugh B and C (CPB-C) had significantly elevated H3Cit-DNA levels as compared to healthy controls and patients with Child-Pugh A (CPA). The difference between Child-Pugh A and healthy controls was not significant. **b)** Patients with Child-Pugh B and C (CPB-C) had significantly elevated MPO-DNA levels as compared to healthy controls and patients with Child-Pugh A (CPA). The difference between Child-Pugh A and healthy controls was not significant.

Figures were created using GraphPad Prism version 5.04 for Windows, GraphPad Software, San Diego, California, USA, www.graphpad.com

Supplementary Table S1

Comparison between HCC patients with Child-Pugh A (CPA) cirrhosis and HCC patients with no cirrhosis regarding H3Cit-DNA, MPO-DNA and TAT.

	**HCC cirrhosis (CPA)** Median (IQR)	**HCC no cirrhosis** Median (IQR)	**P-value**
H3Cit-DNA (ng/mL)	52.1 (5.0 - 119.7)	57.4 (12.6 - 330.5)	0.34
MPO-DNA (O.D.)	0.30 (0.15 - 0.64)	0.21 (0.14 - 0.56)	0.64
TAT (μg/mL)	3.43 (2.44 - 4.81)	3.52 (2.20 - 5.27)	0.84
